# Virtual Photon-Mediated Quantum State Transfer and Remote Entanglement between Spin Qubits in Quantum Dots Using Superadiabatic Pulses

**DOI:** 10.3390/e26050379

**Published:** 2024-04-29

**Authors:** Yue Wang, Ting Wang, Xing-Yu Zhu

**Affiliations:** 1 School of Mechanical and Electronic Engineering, Suzhou University, Suzhou 234000, China; 2 Institute of Quantum Information Technology, Suzhou University, Suzhou 234000, China

**Keywords:** spin qubit, circuit quantum electrodynamics, quantum state transfer, remote entanglement, superadiabatic pulse

## Abstract

Spin qubits in semiconductor quantum dots are an attractive candidate for scalable quantum information processing. Reliable quantum state transfer and entanglement between spatially separated spin qubits is a highly desirable but challenging goal. Here, we propose a fast and high-fidelity quantum state transfer scheme for two spin qubits mediated by virtual microwave photons. Our general strategy involves using a superadiabatic pulse to eliminate non-adiabatic transitions, without the need for increased control complexity. We show that arbitrary quantum state transfer can be achieved with a fidelity of 95.1% within a 60 ns short time under realistic parameter conditions. We also demonstrate the robustness of this scheme to experimental imperfections and environmental noises. Furthermore, this scheme can be directly applied to the generation of a remote Bell entangled state with a fidelity as high as 97.6%. These results pave the way for fault-tolerant quantum computation on spin quantum network architecture platforms.

## 1. Introduction

Electron spins in semiconductor quantum dots have emerged as a promising platform for achieving large-scale quantum information processing, due to their small footprint, long coherence times, and compatibility with advanced semiconductor manufacturing techniques [1,2]. Recently, there have been significant advancements in high-fidelity single-qubit and two-qubit gate operations [3,4,5], as well as in the universal control of multiple-qubit processors [6,7,8]. For scalable quantum computing, it is essential to find a suitable quantum architecture that can leverage the feasible state-of-art technology for integrating large numbers of qubits. A great deal of progress has been made in the monolithic architectures [9,10,11], primarily based on the wavefunction overlap between adjacent quantum dots. In contrast, network architectures [12,13,14], which utilize flying photons to establish links between long-distance qubits, represent a more achievable solution due to their advantages in flexibility and connectivity. However, a critical step in realizing network architectures is to achieve efficient and high-fidelity quantum state transfer and entanglement between spatially separated spin qubits.

Circuit quantum electrodynamics (circuit QED) provides an attractive way to realize quantum network architectures [15,16,17], where a superconducting resonator serves as the quantum bus interacting with different qubits. The typical approach for implementing quantum state transfer is based on real photon processes [18,19], where one qubit sends quantum information to the microwave photons, and then another qubit receives the information from the photons. This indirect approach introduces additional operations and requires fine-tuning of parameters, which results in obstacles to large-scale quantum information processing. In addition, the resonant qubit-resonator coupling leads to additional decoherence processes due to the leakage of resonator photons, thereby affecting the performance of quantum state transfer.

Another attractive approach is to employ virtual photon processes [20,21,22,23,24,25,26], where the frequencies of the two qubits are detuned from the resonator frequency. In this case, the effective coupling between the two qubits allows for the direct transfer of quantum information between them. This direct approach can avoid additional resource consumption and quantum information loss due to photon leakage. However, designing a scheme for long-distance quantum state transfer of spin qubits is a highly desirable but challenging goal. On the one hand, the strong coupling between spin qubit and resonator is a challenge due to the small magnetic dipole moment of spin qubits. On the other hand, quantum state transfer requires fast and high fidelity, even in the presence of operational errors and decoherence effects. Furthermore, quantum entanglement is a crucial resource for quantum computation. Hence, quantum state transfer schemes need to be applicable to remote entanglement preparation. Such a scheme for spin qubits has not yet been demonstrated, as meeting all the implementation requirements mentioned above is not a straightforward task.

In this paper, we propose an efficient quantum state transfer scheme for long-distance spin qubits based on the circuit QED architecture, addressing all the challenges. Firstly, we employ a single-electron spin qubit in a double quantum dot (DQD). Due to spin-charge hybridization, the spin qubit exhibits charge characteristics, enabling strong coupling with the resonator [23,27,28]. Secondly, we design a superadiabatic pulse for state transfer between spin qubits mediated by virtual microwave photons. By modifying the parameters of the control pulse, we not only effectively eliminate non-adiabatic transitions but also significantly reduce the state transfer time. We show that the quantum state transfer can achieve a high fidelity of 95.1% within a short time of 60 ns under realistic conditions, while exhibiting robustness to experimental imperfections and environmental noises. Furthermore, our scheme can be directly applied to the generation of two-qubit remote entanglement on demand with a high fidelity of 97.6%, which already meets the threshold for network error correction protocols [12,13]. These results provide the key elements for scalable spin-based quantum information processing with the network architecture.

## 2. Setup and Models

**Figure 1:**
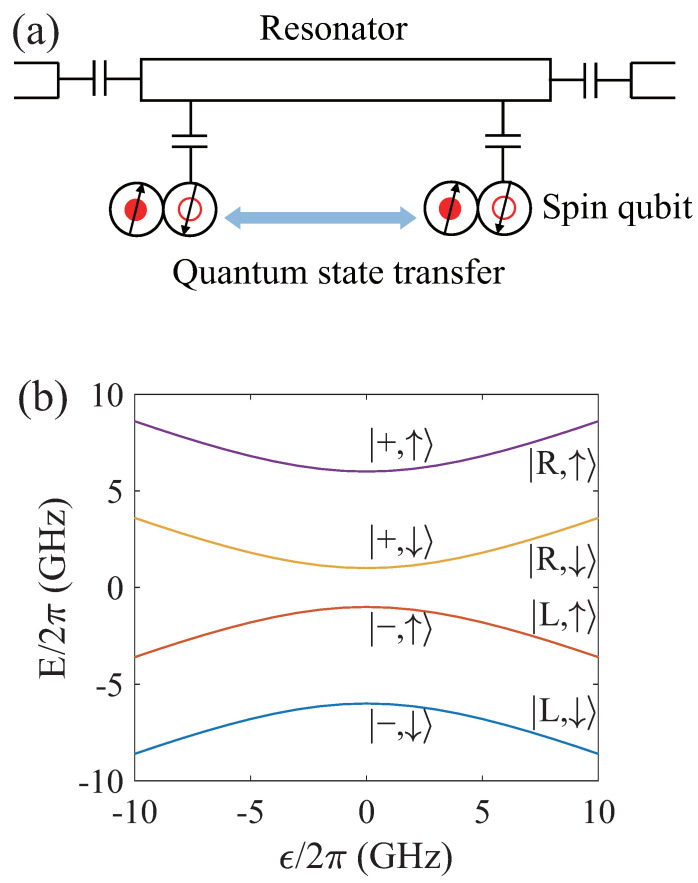
(**a**) Schematic of two-spin-qubit quantum state transfer. A single electron in a double quantum dot is capacitively coupled to a superconducting resonator. The quantum state transfer of two long-distance spin qubits is achieved through the virtual photon process. (**b**) The level structure of the spin qubit as a function of the parameter ϵ. The parameters of the system are as follows: $t_{c}/2\pi =3.5$ GHz, $g\mu_{B}B/2\pi =5.0$ GHz, and $g\mu_{B}\Delta B/2\pi =0.2$ GHz.

We consider two silicon-based semiconductor spin qubits coupled to both ends of the joint superconducting resonator, as illustrated in Figure 1a. Here, a single electron confined in a double quantum dot forms the |L〉 and |R〉 charge states, representing the electron occupying the left or right dot, respectively. The potential difference between two dots is ϵ and the tunneling coupling between two dots is $t_{c}$. By applying a uniform external magnetic field *B*, the electron undergoes Zeeman splitting, forming the |↑〉 and |↓〉 spin states. In addition, a nearby micromagnet generates a gradient magnetic field ΔB between the left and right dots, inducing spin-charge hybridization in the electron [29,30]. The Hamiltonian describing the single-electron DQD is

(1) $$H_{DQD}=\frac{1}{2}\epsilon {\tilde{\tau }}_{z}+t_{c}{\tilde{\tau }}_{x}+\frac{1}{2}g\mu_{B}\left(B{\tilde{\sigma }}_{z}+\Delta B{\tilde{\sigma }}_{x}{\tilde{\tau }}_{z}\right),$$

where g=2 is the electronic Lande g-factor, and $\mu_{B}$ is the Bohr magneton. The operators ${\tilde{\tau }}_{i}$ and ${\tilde{\sigma }}_{i}$ are Pauli operators defined in the charge and spin subspaces, respectively.

In Figure 1b, we show the energy levels of the single-electron DQD system as a function of the bias ϵ. The energy spectrum can be conveniently understood through the following aspects. On one hand, when ϵ is large, the electron is localized in either the left or right dot, corresponding to the states |L,↓〉, |L,↑〉, |R,↓〉, and |R,↑〉. On the other hand, when ϵ=0, the electron is delocalized across the DQD, leading to the bonding and antibonding states $|\mp \rangle =(|R\rangle \mp |L\rangle )/\sqrt{2}$. Furthermore, due to the spin-charge interaction, the bonding and antibonding states with opposite spins |−,↑〉, |+↓〉 are hybridized. We encode the spin qubit using the lowest two energy levels, denoted as |0〉 and |1〉. Thus, the effective two-level Hamiltonian of the spin qubit can be written as

(2) $$H_{s}=\frac{\hbar }{2}\omega_{s}\sigma_{z},$$

where $\omega_{s}$ is the transition frequency of the spin qubit, and the operator $\sigma_{z}$ is the Pauli operator defined in the basis of states |0〉 and |1〉.

Then, we consider a superconducting microwave resonator, neglecting high-energy modes and focusing only on the fundamental mode. The Hamiltonian of the resonator is represented by

(3) $$H_{r}=\hbar \omega_{r}a^{†}a,$$

where $\omega_{r}$ is the resonator frequency, and the operators $a^{†}$ and *a* are the creation and annihilation operators, respectively. When the resonator is capacitively coupled to the spin qubit, it exhibits a charge-resonator coupling strength $g_{c}$. Due to the hybridization of the spin qubit with charge and spin states, the spin and resonator can be coupled by an indirect electric-dipole interaction, with a spin-photon coupling strength $g_{s}=\langle 0|\tau |1\rangle g_{c}$. In this way, the interaction Hamiltonian between the spin qubit and the resonator can be expressed as

(4) $$H_{int}=\hbar g_{s}\left(a^{†}+a\right)\sigma_{x}.$$

To achieve control over the composite system, we apply an external driving field to modulate the frequency of one of the spin qubits, with $H_{d}=\frac{\hbar }{2}f\left(t\right)\sigma_{z}$. Here, f(t) represents the frequency response to the driving pulse and is chosen to satisfy the following adjustable sinusoidal function: $\int_{0}^{t}f\left(\tau \right)d\tau =A\left(t\right)\sin\left[f\left(t\right)t+\beta \left(t\right)\right]$, where A(t) and β(t) are the amplitude and phase of the driving pulse, respectively. Putting things together, in the rotating wave approximation, the composite system comprising both spin qubits and a resonator can be described by the following Hamiltonian

(5) $$H_{com}=\sum_{i=1,2}\frac{\hbar }{2}\omega_{s}^{i}\sigma_{z}^{i}+\hbar \omega_{r}a^{†}a+\sum_{i=1,2}\hbar g_{s}^{i}\left(a^{†}\sigma_{-}^{i}+a\sigma_{+}^{i}\right)+\frac{\hbar }{2}f\left(t\right)\sigma_{z}^{1},$$

where the superscript *i* represents the spin qubits 1 and 2.

## 3. Spin–Spin Coupling Mediated by Virtual Photons

We now consider the effective coupling between spin qubits mediated by virtual photons in the resonator. In the dispersive regime, the frequency detuning between the spin qubit and the resonator is larger than the coupling strength, satisfying $|\Delta_{s}^{i}\left|=\right|\omega_{r}-\omega_{s}^{i}|>g_{s}^{i}$. In this case, the impact of photon excitation on the superposed eigenstates of the two spin qubits is suppressed. Using the Schrieffer–Wolff transformation [31], we decouple different subspaces at a desired order, and the system can be described by the Tavis–Commings Hamiltonian [32]

(6) $$H_{disp}=\sum_{i=1,2}\frac{\hbar }{2}\omega \prime_{s}^{i}\sigma_{z}^{i}+\hbar g_{eff}\left(\sigma_{+}^{1}\sigma_{-}^{2}+\sigma_{-}^{1}\sigma_{+}^{2}\right)+\frac{\hbar }{2}f\left(t\right)\sigma_{z}^{1},$$

where $\omega \prime_{s}=\omega_{s}-g_{s}^{2}/\Delta_{s}$ is the frequency of spin qubit, $\sigma_{\pm }$ are the raising and lowering operators, and the spin–spin effective coupling strength is

(7) $$g_{eff}=\frac{g_{s}^{1}g_{s}^{2}}{2}\left(\frac{1}{\Delta_{s}^{1}}+\frac{1}{\Delta_{s}^{2}}\right).$$

For a more intuitive understanding of the effective interaction between spin qubits, we apply a unitary transformation to transform $H_{disp}$ into a rotating frame, with

(8) $$U_{1}=\exp\left[i\frac{\omega_{s}^{1}t}{2}\sigma_{z}^{1}+i\frac{\omega_{s}^{2}t}{2}\sigma_{z}^{2}+i\frac{\int_{0}^{t}f\left(\tau \right)d\tau }{2}\sigma_{z}^{1}\right].$$

This leads to the effective Hamiltonian

(9) $$H_{eff}=\hbar g_{eff}J_{1}\left[A\left(t\right)\right]\exp\left\{i[\delta (t)t-\beta (t)\right]\}\sigma_{+}^{1}\sigma_{-}^{2}+H.c.$$

Here, we use the Jacobi–Anger expansion $e^{iz\sin\alpha }=\sum_{-\infty }^{\infty }J_{n}\left(z\right)e^{in\alpha }$, $J_{1}\left[A\left(t\right)\right]$ is the first-order Bessel function, and $\delta \left(t\right)=\omega \prime_{s}^{2}-\omega \prime_{s}^{1}-f\left(t\right)$ is the frequency detuning. Furthermore, we apply an extra unitary transformation, with

(10) $$U_{2}=\exp\left[-i\frac{\delta (t)t}{4}\sigma_{z}^{1}+i\frac{\delta (t)t}{4}\sigma_{z}^{2}\right].$$

In the new frame, the effective Hamiltonian can be rewritten as

(11) $$H_{eff}=\frac{\hbar }{2}\left[\begin{array}{cccc} 0 & 0 & 0 & 0\\ 0 & d[\delta (t)t]/dt & 2g_{eff}J_{1}\left[A\left(t\right)\right]e^{-i\beta (t)} & 0\\ 0 & 2g_{eff}J_{1}\left[A\left(t\right)\right]e^{i\beta (t)} & -d[\delta (t)t]/dt & 0\\ 0 & 0 & 0 & 0 \end{array}\right].$$

It is worth noting that we can achieve transitions between the states |01〉 and |10〉 by adjusting the parameters of the driving pulse. For more discussion on the comparison of the effective Hamiltonian with the full Hamiltonian, see Appendix A.

## 4. Superadiabatic Pulse

For convenience, we consider the system Hamiltonian driven by an external driving field in the subspace spanned by the states |01〉 and |10〉, with

(12) $$H_{sub}=\frac{\hbar }{2}\left[\begin{array}{cc} \Delta (t) & \Omega (t)\\ \Omega (t) & -\Delta (t) \end{array}\right],$$

where Δ(t) is the frequency detuning, and Ω(t) is the frequency of the Rabi oscillation. During the evolution of the system, there will be non-adiabatic transitions between the instantaneous eigenstates. The usual strategy is to apply an adiabatic pulse, where the pulse parameters change very slowly over time. While this approach allows the system to evolve along eigenstates, the long evolution time implies more accumulated errors due to environmental noise, thereby limiting the achievement of fast and high-fidelity state transfer.

To address this challenge, we employ a superadiabatic pulse scheme to achieve fast evolution of the system while effectively eliminating non-adiabatic transitions [33,34]. For the time-dependent Hamiltonian $H_{sub}$, the non-adiabatic transition evolution part can be expressed as

(13) $$H_{na}=-i\hbar \sum_{n=\pm }[|\partial_{t}\lambda_{n}\left(t\right)\rangle \langle \lambda_{n}\left(t\right)|-\langle \lambda_{n}\left(t\right)|\partial_{t}\lambda_{n}\left(t\right)\rangle |\lambda_{n}\left(t\right)\rangle \langle \lambda_{n}\left(t\right)|],$$

where $|\lambda_{n}\left(t\right)\rangle$ are the instantaneous eigenstates of $H_{sub}$, and $|\partial_{t}\lambda_{n}\left(t\right)\rangle$ are the derivatives of the instantaneous eigenstates with respect to time. In the subspace of states |01〉 and |10〉, the non-adiabatic transition part can be expressed in a specific form as

(14) $$H_{na}=\frac{\hbar }{2}\left[\begin{array}{cc} 0 & i\dot{\theta }\left(t\right)\\ -i\dot{\theta }\left(t\right) & 0 \end{array}\right],$$

where angle θ(t)=arctan[Ω(t)/Δ(t)]. Our strategy involves starting with the original Hamiltonian $H_{sub}$ and modulating the parameters of the driving field to construct the effective Hamiltonian $H_{eff}$. This effective Hamiltonian satisfies

(15) $$H_{eff}=H_{sub}-H_{na}=\frac{\hbar }{2}\left[\begin{array}{cc} \Delta_{sa}\left(t\right) & \Omega_{sa}\left(t\right)e^{-i\phi_{sa}\left(t\right)}\\ \Omega_{sa}\left(t\right)e^{i\phi_{sa}\left(t\right)} & -\Delta_{sa}\left(t\right) \end{array}\right],$$

where the parameters of the superadiabatic pulse are $\Delta_{sa}\left(t\right)=\Delta \left(t\right)$, $\Omega_{sa}\left(t\right)=\sqrt{\Omega^{2}\left(t\right)+{\dot{\theta }}^{2}\left(t\right)}$, and $\phi_{sa}\left(t\right)=\arctan\left[\dot{\theta }\left(t\right)/\Omega \left(t\right)\right]$.

We note that a shortcut to the adiabatic scheme, introducing an auxiliary field to eliminate non-adiabatic transitions, has been proposed in various systems [35,36,37]. This scheme not only requires the introduction of additional laser or microwave fields, but also demands careful design to implement interaction terms that are not present in the original Hamiltonian. In contrast, our scheme only requires the modulation of driving pulse parameters, effectively avoiding the complexity of experiments and resource consumption. Combining Equations (11) and (15), we can obtain the relevant parameters for the superadiabatic pulse as

(16) $$\begin{array}{cc} \; & f\left(t\right)=\omega \prime_{s}^{2}-\omega \prime_{s}^{1}-\frac{\int_{0}^{t}\Delta \left(\tau \right)d\tau }{t}\\ \; & A\left(t\right)=J_{1}^{-1}\left\{\frac{1}{2g_{eff}}\sqrt{\Omega^{2}\left(t\right)+\frac{{\left[\Delta \left(t\right)\dot{\Omega }\left(t\right)-\Omega \left(t\right)\dot{\Delta }\left(t\right)\right]}^{2}}{{\left[\Delta^{2}\left(t\right)+\Omega^{2}\left(t\right)\right]}^{2}}}\right\}\\ \; & \beta \left(t\right)=\arctan\left\{\frac{\Delta \left(t\right)\dot{\Omega }\left(t\right)-\Omega \left(t\right)\dot{\Delta }\left(t\right)}{\Omega \left(t\right)[\Delta^{2}\left(t\right)+\Omega^{2}\left(t\right)]}\right\}. \end{array}$$

We set the parameters to satisfy the condition $\sqrt{\Omega^{2}\left(t\right)+{\dot{\theta }}^{2}\left(t\right)}\leq 2gJ_{1}^{\max}$, where $J_{1}^{\max}=0.582$ is the maximum value of the first-order Bessel function.

## 5. Quantum State Transfer

**Figure 2:**
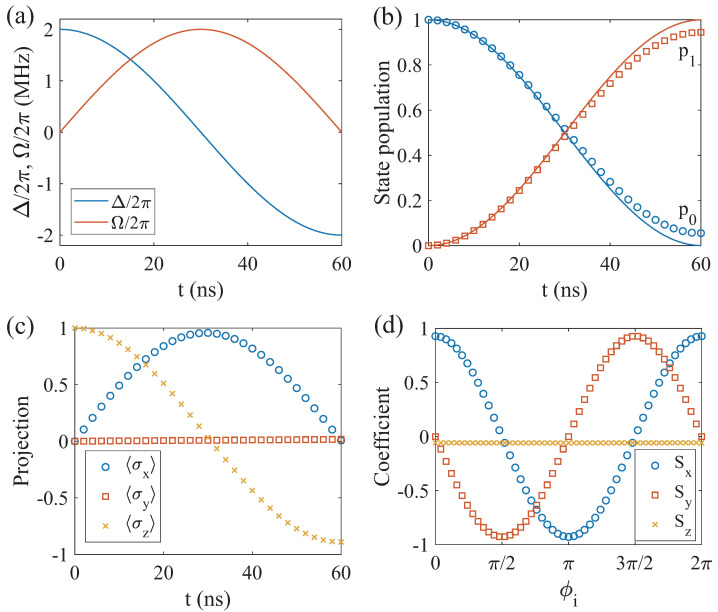
(**a**) The scheme for implementing the quantum state transfer by time-dependent parameters of the driving pulse. (**b**) The state populations of spin qubit 2 during the quantum state transfer process, with spin qubit 1 and 2 initially in |1〉 and |0〉. The solid line is the evolution without environmental noises and the dotted line is the evolution with environmental noises. (**c**) The quantum state tomography of spin qubit 2 involves projecting the state onto the Pauli matrices $\sigma_{x}$, $\sigma_{y}$ and $\sigma_{z}$. (**d**) The coefficients $S_{x}$, $S_{y}$ and $S_{z}$ of the states of spin qubit 2 as a function of initial phase $\phi_{i}$ in the quantum state transfer, with spin qubit 1 initially in $(|0\rangle +e^{i\phi_{i}}\left|1\rangle \right)/\sqrt{2}$.

Having achieved effective spin–spin coupling mediated by virtual photons, we can use the superadiabatic pulse scheme for quantum state transfer between two distant spin qubits. As shown in Figure 2a, we set the typical time-dependent parameters of the driving field as

(17) $$\begin{array}{c} \Omega \left(t\right)=\Omega_{0}\sin\left(\frac{\pi t}{T}\right)\\ \Delta \left(t\right)=\Delta_{0}\cos\left(\frac{\pi t}{T}\right), \end{array}$$

where *T* is the duration time, $\Omega_{0}$ and $\Delta_{0}$ are the maximum values of the Rabi oscillation frequency and the frequency detuning, respectively. This pulse has two advantages over other types of pulses, such as the Gaussian pulse: its simple waveform and no need for cutoff.

The evolution of the composite system is described by the master equation [15,38]

(18) $$\frac{d\rho }{dt}=\frac{i}{\hbar }\left[\rho ,H_{eff}\right]+\sum_{i=1,2}\left(\gamma_{1}^{i}+\frac{g_{s}^{i2}\kappa }{\Delta_{s}^{i2}}\right)D\left[\sigma_{-}^{i}\right]\rho +\sum_{i=1,2}\frac{\gamma_{\phi }^{i}}{2}D\left[\sigma_{z}^{i}\right]\rho ,$$

where ρ is the density matrix of the system, and $D\left[O\right]\rho =O\rho O^{†}-\left(O^{†}O\rho +\rho O^{†}O\right)/2$ is the Lindblad operator describing the decoherence processes. $\gamma_{1}=1/T_{1}$ and $\gamma_{\phi }=1/T_{\phi }$ are the relaxation and dephasing rates of the spin qubit. κ is the leakage rate of photons in the resonator. For more on the derivation of the effective master equation, see Appendix B. Here, we use the parameter values from the experiments [23,27,39]: spin-resonator coupling strength $g_{s}^{1(2)}/2\pi =40$ MHz, frequency detuning $\Delta_{s}^{1(2)}=5g_{s}^{1(2)}$, relaxation time $T_{1}^{1(2)}=1.2$ ms, dephasing time $T_{\phi }^{1(2)}=1\;\mu$s, leakage rate κ/2π=1.8 MHz, pulse parameters $\Omega_{0}/2\pi =\Delta_{0}/2\pi =2$ MHz, duration time T=60 ns.

To study the quantum state transfer from spin qubit 1 to spin qubit 2, we first prepare the initial states of spin qubit 1 and 2 as |1〉 and |0〉, respectively. Then, a superadiabatic pulse is applied to drive spin qubit 1 and 2 to evolve towards the |0〉 and |1〉 states, respectively. Finally, we present the state population of spin qubit 2 during the state transfer process. As shown in Figure 2b, solid and dotted lines represent the ideal case and the practical case including environmental noises, respectively. We observe a smooth increase in the population of state |1〉, which ultimately stabilizes at $p_{1}=94.5\%$, indicating the efficiency of the state transfer using this scheme. In Figure 2c, we obtained the state evolution trajectory of spin qubit 2 by projecting the state onto three different orthogonal basis vectors $\sigma_{i=x,y,z}$ and calculating three components $\langle \sigma_{i=x,y,z}\rangle$, where σ represents the Pauli operator. These results demonstrate that the proposed scheme can achieve quantum state transfer between two spin qubits.

The phase of quantum states is crucial in quantum information processing, so we investigate the preservation of phase information during quantum state transfer. We initialize spin qubit 1 in a superposition state $|\psi_{i}\rangle =(|0\rangle +e^{i\phi_{i}}\left|1\rangle \right)/\sqrt{2}$, and implement the same quantum state transfer process. In the ideal case, the final state of spin qubit 2 should be $|\psi_{f}\rangle =(|0\rangle +e^{i\phi_{f}}\left|1\rangle \right)/\sqrt{2}$, where the phase $\phi_{f}=\phi_{i}$. In practice, however, the phase information is affected by the environmental noises. We simulate the quantum state transfer process and obtain the final state of spin qubit 2, which can be represented as $\rho =(S_{I}I+S_{x}\sigma_{x}+S_{y}\sigma_{y}+S_{z}\sigma_{z})/2$. In Figure 2d, we show the coefficients $S_{x},S_{y},S_{z}$ as a function of the initial phase $\phi_{i}$, indicating that phase coherence is maintained during the transfer process.

**Figure 3:**
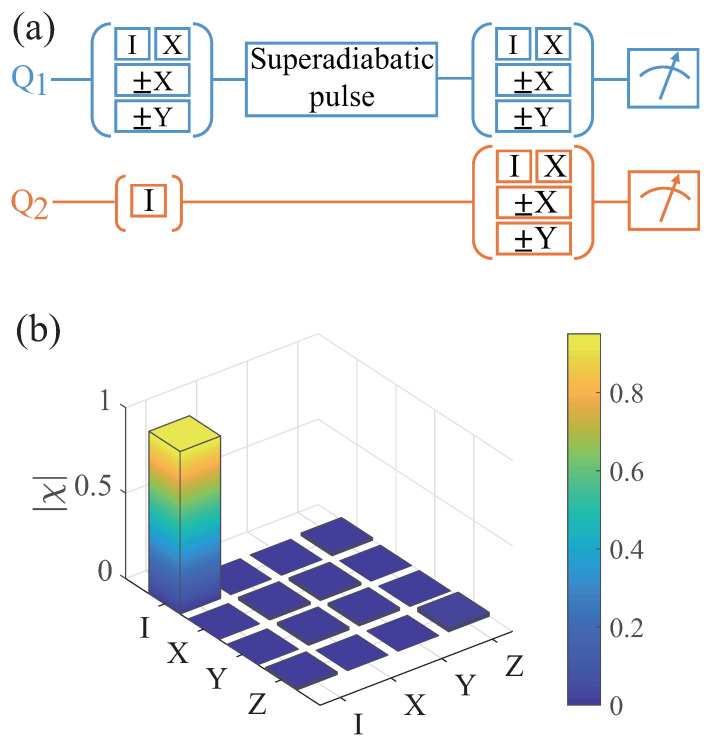
(**a**) The whole pulse sequence is used to characterize the quantum state transfer between the two spin qubits. We prepare six mutually unbiased states of spin qubit 1, while spin qubit 2 is in state |0〉. Next, we apply a superadiabatic pulse to transfer the state of spin qubit 1 to spin qubit 2. Finally, we obtain the quantum state tomography of the system through joint readout. (**b**) The process matrix of the state transfer in the basis {I,X,Y,Z} using the quantum process tomography, and the fidelity reaches F=95.1%.

To quantitatively evaluate the quantum state transfer between distant spin qubits using our scheme, we calculate the process fidelity using quantum process tomography (QPT) [40]. As shown in Figure 3a, we prepare spin qubit 1 in the six mutually unbiased states |0〉, |1〉, $(|0\rangle \pm \left|1\rangle \right)/\sqrt{2}$ and $(|0\rangle \pm i|1\rangle )/\sqrt{2}$, while spin qubit 2 is in state |0〉. Then, we transfer the state of spin qubit 1 to spin qubit 2, and reconstruct the transfer process matrix χ using quantum process tomography. As shown in Figure 3b, the process fidelity for quantum state transfer reaches $F=tr(\chi \chi_{id})=95.1\%$, compared to the ideal transfer process matrix $\chi_{id}$. This indicates our ability to achieve high-fidelity quantum state transfer, with potential applications in remote quantum entanglement.

## 6. Robustness to Imperfections and Noises

**Figure 4:**
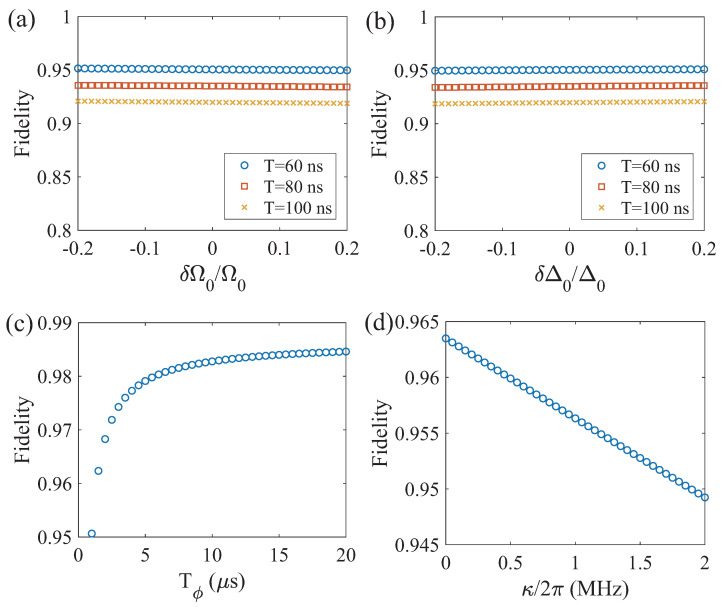
The process fidelity of quantum state transfer as functions of fluctuations in pulse parameters (**a**) $\delta \Omega_{0}/\Omega_{0}$ and (**b**) $\delta \Delta_{0}/\Delta_{0}$. Here, three driving pulses with different duration times are presented. (**c**) The process fidelity of quantum state transfer as a function of the spin qubit dephasing time $\gamma_{\phi }$. (**d**) The process fidelity of quantum state transfer as a function of the photon leakage κ in the resonator.

Given the inevitability of experimental imperfections and environmental noise in practical settings, robustness is a crucial characteristic of quantum state transfer schemes. We investigate the robustness of quantum state transfer to fluctuations in the pulse parameters of our scheme. The performance of quantum state transfer is determined by two key parameters: the Rabi oscillation frequency Ω(t) and the frequency detuning Δ(t), corresponding to the amplitude and frequency of the driving pulse, respectively. Therefore, the fluctuations in the pulse parameters significantly affect the process fidelity. We test the robustness of the state transfer process to parameter fluctuations by adding fluctuations to both the Rabi oscillation frequency and frequency detuning, as $\Omega_{0}\to \Omega_{0}+\delta \Omega_{0}$ and $\Delta_{0}\to \Delta_{0}+\delta \Delta_{0}$. Figure 4a,b show the process fidelity as functions of parameter fluctuations $\delta \Omega_{0}/\Omega_{0}$ and $\delta \Delta_{0}/\Delta_{0}$ for various duration times. We observe that as the duration time increases, the process fidelity decreases. This is because longer duration times imply more error accumulation due to environmental noises. In addition, it is evident that the quantum state transfer process using our scheme exhibits robustness to parameter fluctuations over a wide range.

Furthermore, we investigate the influence of environmental noises on the quantum state transfer process. In silicon-based spin qubits, the dephasing process is considered to be the primary factor limiting the state transfer process, since the relaxation time is much longer compared to the dephasing time. In Figure 4c, we observe that the process fidelity increases with the dephasing time $T_{\phi }$. For instance, when the dephasing time $T_{\phi }$ changes from 1 μs to 20 μs, the process fidelity rapidly increases from 95.1% to nearly 99%. This is attributed to the fact that in our scheme, the coupling between spin qubits is mediated by virtual photons, and the decoherence effects of the spin qubits determine the fidelity of the state transfer process. In addition, the leakage of photons in the resonator can also induce decoherence channels for spin qubits, known as the Purcell effect [15]. In Figure 4d, we observe a gradual increase in process fidelity as the photon leakage rate κ decreases. These results indicate that the process fidelity of the quantum state transfer can be further improved in the future by extending the dephasing time $T_{\phi }$ of spin qubits [41,42] and reducing the photon leakage rate κ in the resonator [43]. For example, when $T_{\phi }=20\;\mu$s and κ/2π=1 MHz, a quantum state transfer with fidelity exceeding 99% should be achievable.

## 7. Generation of Remote Entanglement

**Figure 5:**
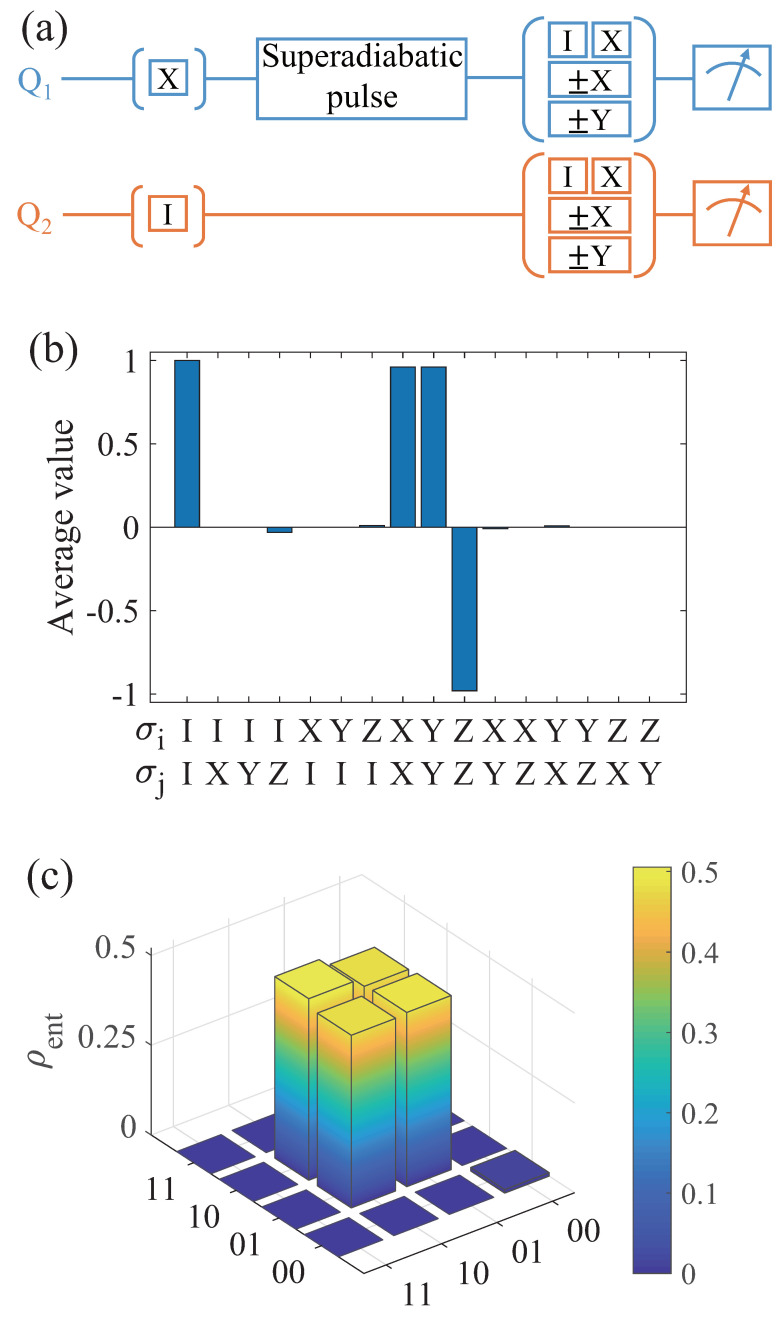
(**a**) The whole pulse sequence is used to generate remote entangled states between spin qubits using our scheme. The spin qubits 1 and 2 can initially be in states |1〉 and |0〉, and then we perform a well-designed superadiabatic pulse. Finally, we obtain the density matrix of the system through quantum state tomography. (**b**) The average value of the Pauli operators $\langle \sigma_{i}\sigma_{j}\rangle$ for the spin qubits. (**c**) The density matrix $\rho_{ent}$ of the generated remote entangled state and the state fidelity is $F_{ent}=97.6\%$ relative to the ideal Bell state.

The generation of remote entanglement between arbitrary qubits is a critical element in network quantum information processing. Here, we utilize the quantum state transfer scheme to generate two-qubit remote entangled states between spin qubits. As shown in Figure 5a, we initially prepare spin qubits 1 and 2 in states |1〉 and |0〉, respectively. Then, a designed driving pulse $R^{\pi /2}$ is applied to spin qubit 1 to generate the entangled Bell state $|\Psi^{+}\rangle =(|01\rangle +|10\rangle )/\sqrt{2}$, which can be achieved by setting the duration time to T=30 ns. Finally, the density matrices of the two qubits can be extracted using quantum state tomography.

Taking into account the decoherence of spin qubits and photon leakage, we numerically simulate the dynamical evolution of the entire system using the master equation. Then, the calculated two-qubit density matrix $\rho_{ent}$ is represented using quantum state tomography. In Figure 5b,c, we show the average values of the Pauli operators $\langle \sigma_{i}\sigma_{j}\rangle$ for the two spin qubits, respectively, and the reconstructed density matrix of the remote Bell entangled state. Compared to the ideal Bell entangled state, our scheme achieves an entanglement fidelity of $F_{ent}=\langle \Psi^{+}|\rho_{ent}|\Psi^{+}\rangle =97.6\%$, exceeding the threshold for many network architecture-based quantum error correction protocols [12,13]. Overall, our scheme is capable of generating remote entangled states between two spin qubits, making it promising for various quantum information processing applications in a network architecture.

## 8. Conclusions

We propose a scheme to achieve fast and high-fidelity quantum state transfer between distant spin qubits. Since the frequency of the spin qubit can be adjusted by an external driving pulse, the interaction between spin qubits can be achieved through virtual photon processes in the dispersive regime. By utilizing the superadiabatic pulse, we achieved the state transfer with high fidelity of 95.1% within 60 ns under the present experimental parameters. Furthermore, we investigated the robustness of this scheme against experimental imperfections and environmental noises. Additionally, our scheme can be directly applied to generate remote entangled states with high performance. Such quantum state transfer and remote entanglement are crucial quantum resources that can enhance the connectivity and flexibility of network architectures, making them more suitable for the implementation of quantum error correction protocols and fault-tolerant quantum computing. Therefore, this scheme provides a powerful solution for quantum information processing in spin-based architectures and can be extended to various solid-state quantum systems in the future.

## Figures and Tables

**Figure A1:**
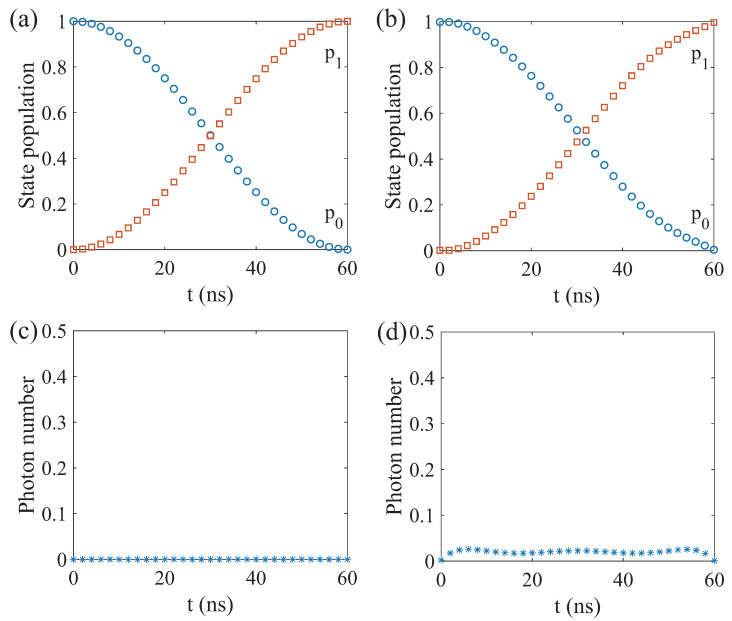
The state population of spin qubit 2 during the quantum state transfer process is obtained using (**a**) the effective Hamiltonian and (**b**) the full Hamiltonian. (**c**,**d**) correspond to the remaining excited photon number in the resonator, respectively. Here, we neglect the influence of the environmental noises.

## Data Availability

The data that support the findings of this study are available from the corresponding author upon reasonable request.

## Appendix A. Comparison of Effective Hamiltonian and Full Hamiltonian

In this paper, we consider two spin qubits coupled to the same resonator, and obtain the effective spin–spin Hamiltonian $H_{eff}$ in the dispersive regime, as given by Equation (11). Compared to the full Hamiltonian $H_{com}$ of the system given by Equation (5), the effective Hamiltonian removes the resonator photon term and only involves the two spin qubits in the subspace {01,10}. To validate the accuracy of our model, we simulate the quantum state transfer process between two spin qubits using both the effective Hamiltonian and the full Hamiltonian. For simplicity, we ignore the influence of the environmental noises.

As shown in Figure A1a,b, we calculate the population dynamics of spin qubit 2 over time. These are obtained by using the effective Hamiltonian and the full Hamiltonian, respectively. In Figure A1a, the population of state |1〉 increases smoothly, eventually reaching $p_{1}=100\%$, indicating a perfect quantum state transfer between the two spin qubits. In Figure A1b, the population of state |1〉 stabilizes at $p_{1}=99.6\%$. This is because the full Hamiltonian used includes the resonator term. During the quantum state transfer process, there are a few excited photons present in the resonator, which hinders the perfect quantum state transfer between two spin qubits. Figure A1c,d represent the average photon number in the resonator during the quantum state transfer process, corresponding to the effective Hamiltonian and the full Hamiltonian, respectively. In Figure A1c, we set the photon number in the resonator to remain at 0. In Figure A1d, we observe that there are a few excited photons, with the photon number ranging from 0 to 0.026, consistent with our previous analysis.

Upon comparison, we find that within the selected parameter range, the influence of resonator photons on the quantum state transfer process is only ∼0.4%. Therefore, we can confidently ignore the impact of photon excitations on the superposed eigenstates of the two spin qubits. These results indicate that the two-spin qubit model obtained through approximation is appropriate.

## Appendix B. The Derivation of the Effective Master Equation

For a spin-resonator coupled system, it can be described using the Jaynes–Cummings Hamiltonian

(A1) $$H_{JC}=\frac{\hbar }{2}\omega_{s}\sigma_{z}+\hbar \omega_{r}a^{†}a+\hbar g_{s}\left(a^{†}\sigma_{-}+a\sigma_{+}\right),$$

where $\omega_{s}$ is the spin qubit frequency, $\omega_{r}$ is the resonator frequency, and $g_{s}$ is the spin-resonator coupling strength. Diagonalizing the above Hamiltonian, we can obtain the excited eigenstates (dressed states)

(A2) $$\begin{array}{cc} {|\psi \rangle }_{+,n} & =\cos\theta_{n}|1,n\rangle +\sin\theta_{n}|0,n+1\rangle \\ {|\psi \rangle }_{-,n} & =-\sin\theta_{n}|1,n\rangle +\cos\theta_{n}|0,n+1\rangle , \end{array}$$

where |0〉 and |1〉 are the ground state and excited state of the spin qubit, while |n〉 is the number of photons in the resonator. The mixing angle is $\theta_{n}=\frac{1}{2}\arctan\left(\frac{2g_{s}\sqrt{n+1}}{\Delta_{s}}\right)$, and the spin-resonator detuning is $\Delta_{s}=\omega_{s}-\omega_{r}$.

In the dispersive regime, which satisfies the condition of large detuning $\Delta_{s}\gg g_{s}$, the eigenstates of the one excitation manifold have the following form

(A3) $$\begin{array}{cc} {|\psi \rangle }_{+,0} & ∼|1,0\rangle +\frac{g_{s}}{\Delta_{s}}|0,1\rangle \\ {|\psi \rangle }_{-,0} & ∼-\frac{g_{s}}{\Delta_{s}}|1,0\rangle +|0,1\rangle . \end{array}$$

The corresponding decay rate can be simply expressed as

(A4) $$\begin{array}{cc} \Gamma_{+,0} & ≃\gamma +{\left(\frac{g_{s}}{\Delta_{s}}\right)}^{2}\kappa \\ \Gamma_{-,0} & ≃{\left(\frac{g_{s}}{\Delta_{s}}\right)}^{2}\gamma +\kappa , \end{array}$$

where γ is the decoherence rate of spin qubits, and κ is the leakage rate of photons. It is easy to see that for spin qubits, besides the inherent decoherence process, photon loss also introduces an additional leakage channel, known as the Purcell effect.

In the presence of the environmental noises, the effective Hamiltonian for the system given by Equation (11) becomes

(A5) $$H_{tot}=H_{eff}+\sum_{i=1,2}\left(H_{\gamma_{1}^{i}}+H_{\gamma_{\phi }^{i}}+H_{\gamma_{\kappa }^{i}}\right),$$

where $H_{\gamma_{1}}$, $H_{\gamma_{\phi }}$ and $H_{\gamma_{\kappa }}$ represent the spin qubit relaxation process, dephasing process, and the Purcell effect process, respectively. In this case, the evolution of the system can be described by the von Neumann equation

(A6) $$\frac{d\rho_{tot}}{dt}=\frac{i}{\hbar }\left[\rho_{tot},H_{tot}\right].$$

When the coupling between the system and the environment is weak, the reduced density matrix of the system follows the master equation under the Markovian approximation.

(A7) $$\frac{d\rho }{dt}=\frac{i}{\hbar }\left[\rho ,H_{eff}\right]+\sum_{i=1,2}\left(\gamma_{1}^{i}+\frac{g_{s}^{i2}\kappa }{\Delta_{s}^{i2}}\right)D\left[\sigma_{-}^{i}\right]\rho +\sum_{i=1,2}\frac{\gamma_{\phi }^{i}}{2}D\left[\sigma_{z}^{i}\right]\rho ,$$

where $\gamma_{1}$ and $\gamma_{\phi }$ are the relaxation rate and dephasing rate of spin qubit, and $\frac{g_{s}^{2}\kappa }{\Delta_{s}^{2}}$ is the decay rate of spin qubit due to the photon leakage. This is the Equation (18) in the main text.
